# Effect of Early Peptide Diets on Zebrafish Skeletal Development

**DOI:** 10.3390/biom13040659

**Published:** 2023-04-07

**Authors:** Alice Printzi, George Koumoundouros, Vincent Fournier, Lauriane Madec, Jose-Luis Zambonino-Infante, David Mazurais

**Affiliations:** 1 Biology Department, University of Crete, 70013 Crete, Greece; 2 IFREMER, University of Brest, CNRS, IRD, LEMAR, F-29280 Plouzané, France; 3 DianaAqua, Symrise Group, 56250 Elven, France

**Keywords:** nutrition, peptides, skeletal development, haemal lordosis, zebrafish, gene markers

## Abstract

Incorporation of dietary peptides has been correlated with decreased presence of skeletal abnormalities in marine larvae. In an attempt to clarify the effect of smaller protein fractions on fish larval and post-larval skeleton, we designed three isoenergetic diets with partial substitution of their protein content with 0% (C), 6% (P6) and 12% (P12) shrimp di- and tripeptides. Experimental diets were tested in zebrafish under two regimes, with inclusion (ADF-Artemia and dry feed) or lack (DF-dry feed only) of live food. Results at the end of metamorphosis highlight the beneficial effect of P12 on growth, survival and early skeletal quality when dry diets are provided from first feeding (DF). Exclusive feeding with P12 also increased the musculoskeletal resistance of the post-larval skeleton against the swimming challenge test (SCT). On the contrary, Artemia inclusion (ADF) overruled any peptide effect in total fish performance. Given the unknown species’ larval nutrient requirements, a 12% dietary peptide incorporation is proposed for successful rearing without live food. A potential nutritional control of the larval and post-larval skeletal development even in aquaculture species is suggested. Limitations of the current molecular analysis are discussed to enable the future identification of the peptide-driven regulatory pathways.

## 1. Introduction

Fish skeletal development is under the continuous influence of biotic and abiotic parameters [1]. Appearance of skeletal deformities, either as distortions of the normal developmental processes (e.g., embryonic and larval stages [2,3]) or as deformations of initially normally developed bones (e.g., post-larval stages [4,5]) is correlated with reduced growth, survival, morphological quality and disrupted general performance (e.g., swimming, susceptibility to diseases [6]). Being a valuable welfare index for both aquaculture and laboratory animals [7,8], skeletal abnormalities appearance entails the quality of the experimental results and, inevitably, the marketability of the aquaculture products.

One of the most critical factors towards fish normal skeletal development is appropriate larval nutrition [9]. Unbalanced or unfavourable provision of macro and micronutrients (e.g., proteins, lipids, vitamins, minerals) can affect several biological processes related to skeletogenesis in both marine and freshwater species and increase the abnormalities frequency [10,11,12]. Inclusion level of HUFAs on the diets has been proven critical for osteoblast differentiation through the regulation of key genes involved in skeletal development (*igf1*, *bmp4*, *pparγ* and *bglap*) in European sea bass larvae, *Dicentrarchus labrax* [13]. Regulation of calcium entry in the cells (phospholipids [14]), acceleration of bone formation and cartilage degradation (vitamin A [15]) and regulation of mineralisation (phosphorus [16], vitamin D [17]) have been reported to be influenced by nutrients in several species. Simultaneously, dietary protein fraction, being the major component of fishmeal, has also attracted great attention in terms of both essential amino acid and peptide supply [18,19]. A tryptophan deficiency has been proposed to induce muscular fractions leading to increased scoliosis incidents in salmonids [20], whereas a peptide-enhanced digestive maturation in sea bass larvae has also been proposed to favour the harmonious larval development [21].

While exploring early larval nutrient requirements, a switch from live-prey to inert diets was necessary in an attempt to decrease the nutrient variability induced by the live food and reduce the production cost [22]. Key to a successful early weaning is the development of starter diets adjusted to the species requirements with enhanced digestibility and constant nutritional value [23]. Protein hydrolysates were introduced as a more efficient method of digestible protein provision [24], enhancing larval growth, survival, immune function and skeletal development [25,26]. Numerous trials have been conducted to vary protein sources, hydrolysis levels and incorporation levels in larval feeds. A hydrolysate incorporation around 10% of the dry diet has been described as optimum up to now for sea bass and sea bream larvae [25,27,28]. Interestingly, the partial replacement of native fishmeal proteins by di- and tripeptides in sea bass larvae also resulted in a decrease in total malformation frequency [21] against a standard live-prey feeding regime. Through these alternative protein fractions, the necessity of indispensable free amino-acid requirements during early larval stages was also highlighted [29].

Given the direct link between normal bone development and early larvae nutrition, lack of zebrafish nutritional control has led in scarce knowledge over its nutrient requirements increasing the experimental bias [30]. Zebrafish bone development and ossification process share common characteristics with higher vertebrates, revealing a valuable model species for skeletogenesis and deformities research [31,32]. Following the mammalian pattern of osteoblast differentiation, zebrafish express several differentiation markers such as *bglap*, *sparc*, collagens and other bone matrix genes during bone development [33]. Simultaneously, several key genes (e.g., *tgfb*, *foxo1*, *tnni2*, *pparγ*) have been highlighted as differentially regulated behind a musculoskeletal remodeling of normally developed post-larvae bones on the species [34]. Although the digestive enzymatic activity (e.g., *amy2a*, *prss1*, [35]) and nutrient transport (e.g., pept1, [36]) during zebrafish ontogenetic development have been described, the precise nutritional needs remain unrevealed. Concerning the dietary protein requirements for example, different sources, qualities and concentrations have been tested suggesting a ratio around 37.6–44.8% for optimal juvenile growth [37,38,39]. However, nutrient requirements are subject to changes according to the different developmental stages [40,41].

Therefore, although incorporation of peptides in early larvae diets is promising in terms of improving the normal skeletal development, the incorporation rates and the regulatory pathways underlying their beneficial effects have not yet been clarified. The purpose of the present study is to evaluate the effect of two experimental peptide diets (P6, P12) against a control one (C) on the prevention of skeletal deformities on zebrafish larvae and early juveniles. Two feeding regimes with inclusion or lack of live food were applied, due to the controversial background on Artemia incorporation for the normal skeletal development of the species [8,42]. Evaluation was based on (a) the frequency of the skeletal deformities at the end of the larval stage and (b) on the frequency of the swimming-induced lordosis, assessing the ability of a normal vertebral column to withstand excess of swimming conditions. The swimming challenge test is an efficient means of inducing increased mechanical loads, which is the main causative factor for the appearance of haemal lordosis on the post-larvae stages [5,43]. Lastly, the possible effect of the diets on gene markers of larval maturation (digestion, ossification and mineralization) was also examined.

## 2. Materials and Methods

### 2.1. Experimental Diets and Animals

Three isonitrogenous and isoenergetic experimental diets were formulated by partial substitution of the total protein source (fishmeal, CPSP 90) with 0% (C, Control group), 6% (P6) and 12% (P12) shrimp di and tripeptides (Table 1). Control diet (C) was designed according to Cahu et al. (2003) [44] and verified for the appropriateness for zebrafish rearing by Antinero et al. (2023) [45]. All the diets were manufactured at Ifremer-Center of Brittany (Plouzané, France). CPSP 90 and the shrimp peptides were provided by Symrise Aqua Feed (Elven, France).

**Figure 1:**
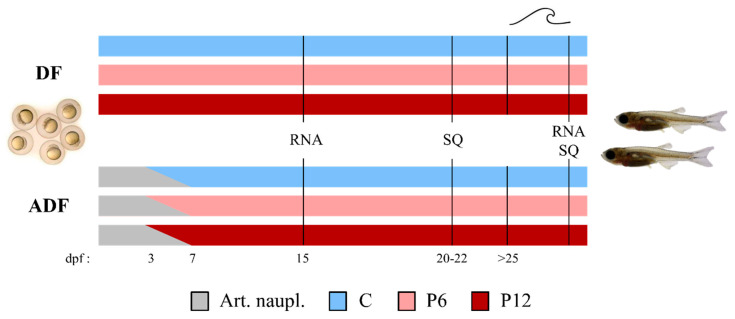
Basic experimental design. Larval rearing was performed under two feeding regimes including (DF) or lacking (ADF) live food supplementation. Three experimental diets (C—Control, P6—6% of dietary peptides and P12—12% of dietary peptides) were tested in triplicate under each feeding regime. Samplings for RNA analysis (RNA) and larval skeletal quality (SQ) took place before and after the swimming challenge test (SCT—marked as a wave symbol). dpf, days post fertilization. Art. naupl., Artemia nauplii.

Two feeding regimes, DF (dry feed only) and ADF (Artemia nauplii and dry feed), each consisting of three experimental replicates, were applied (Figure 1). For each replicate, 3 (C, P6, P12) cubic net pens (4.5 L volume, 100 μm mesh size) were placed inside one 100 L aquarium. Each net pen contained 300 zebrafish embryos derived from a common brood stock for each regime. Aquariums were equipped with a closed recirculation system allowing water circulation. Common abiotic conditions were applied in all treatments and monitored on a daily (temperature, oxygen, pH, conductivity) or weekly (nitrogen compounds) basis (Supplementary Table S1).

In the DF case, larvae were fed five times per day with the experimental diets, from mouth opening until the end of the trial (25–30 dpf, Supplementary Table S2). In the ADF case, a 3-day-exclusive provision of newly hatched Artemia nauplii followed by a 4-day-cofeeding were introduced before a gradual transfer to the experimental dry diets according to Printzi et al. (2021) [8] (Supplementary Table S2).

### 2.2. Sampling and Analytical Methods

Once all the skeletal elements of the zebrafish early juveniles appeared (9–11 mm TL—Total Length, [46]), a random sample of 42–67 individuals was acquired from each population and replicate to evaluate the skeletal deformities rate (number of sampled individuals from each experimental diet in DF: 48–53 repl. 1, 55–67 repl. 2, 42–48 repl. 3 and in ADF: 42–45 repl. 1, 44–46 repl. 2, 48–51 repl. 3). Subsequently, fish were euthanized with an overdose of phenoxyethanol (2-phenoxyethanol, 0.5 mL L^−1^), photographed using a stereoscope (Olympus SZ61) and measured for TL by means of tpsDig2 software [47]. Fixation in phosphate buffered formalin was introduced before the staining for bone and cartilage [48].

When reaching 11.5–12.5 mm TL, 25–40 individuals per population and replicate with no external signs of axial malformations were subjected to the swimming challenge test (SCT) to check the vulnerability of the vertebral column against a prolonged swimming activity. SCT was performed according to Printzi et al. (2021) [5] towards a continuous water velocity of 8.0 TL·s^−1^ for a four-day period. Utilization of three swimming channels (70 cm length, 10 cm depth, 5 cm width) allowed the simultaneous SCT of the three groups of each replicate. During this period, small current cut-offs (10–15 min) enabled the feeding of the individuals according to their regime. At the end of the swimming exercise, fish were euthanized (overdose of 2-phenoxyethanol, 0.5 mL·L^−1^) and photographed using a stereoscope (Olympus SZ61). This allowed the categorization of their external morphology into normal (N) and lordotic (S). The categorization was based on the presence of the characteristic dorsal shift of the caudal peduncle in lordotic (S) group, whereas fish lacking this shift were considered normal (N). After the categorization, fish were sampled either for RNA analysis or stained according to Walker and Kimmel (2007). During the SCT, abiotic parameters were maintained at 28.0 °C (±0.5 °C), 500–700 μS·cm^−1^ conductivity, 7.0–7.5 pH, >90% oxygen saturation and 14/10 h light/dark photoperiod.

Samples for RNA analysis were collected at 15 dpf and after the SCT and preserved in RNA Stabilization Reagent (RNAlater, Qiagen, Hilden, Germany). At 15 dpf, 15 individuals were sampled randomly from each diet, replicate and regime to evaluate the expression of selected genomic markers of larval development in whole larvae samples by real-time PCR (RT-qPCR). The 15 larvae were pooled in 3 samples per diet, replicate and regime (5 individuals/sample) to reach the necessary quantity of dry tissue to follow the analytical procedure described by Mazurais et al. (2008) [10]. Similarly, after SCT, 4–8 individuals of each external phenotype (S, N), experimental diet, replicate and feeding regime were randomly sampled for RNA analysis. A dissection of the haemal parts of the SCT fish was applied according to Printzi et al. 2022 [34]. SCT samples were pooled in two individuals per sample within each phenotype, diet, replicate and regime to meet the extraction requirements (two individuals/sample [10]). Briefly, the total RNA was extracted by Extract-All reagent (Eurobio, Courtaboeuf, Essonne, France) and Nucleospin RNA column (Macherey–Nagel, Düren, Germany). A ND-1000 NanoDrop^®^ spectrophotometer (Thermo Scientific Inc., Waltham, MA, USA) and an Agilent Bioanalyzer 2100 (Agilent Technologies Inc., Santa Clara, CA, USA) verified the concentration/purity and integrity of the samples, respectively. All samples presented an RNA integrity (RIN) score  >  9. After a reverse transcription in duplicate per sample, quantitative PCR analyses for each selected gene were performed. For each target (*bglap*—osteocalcin, pept1—peptide transporter 1a, *amy2a*—amylase alpha 2A, *prss1*—trypsin, *ppargc1a*—peroxisome proliferator-activated receptor gamma coactivator 1 alpha, *ostn*—osteocrin, *tnni2a*—troponin I type 2a, *spp1*—osteopontin, *ihha*—Indian hedgehog signaling molecule a, *foxo1a*—forkhead box O1 a, *tgfb1b*—transforming growth factor beta 1b) and reference genes (*ef1*—eukaryotic translation elongation factor 1 alpha 1 like 1, *actn2b*-actinin alpha 2b), the specific primers designed are listed in the Supplementary Table S3. *Ef1* and *actn2b* were used as reference genes since they did not present any significant differences in their expression between the groups of interest (M values < 0.5).

Survival was estimated at the end of the trials (25–30 dpf) after counting the remaining individuals.

### 2.3. Analysis of the Experimental Diets

Analysis of the peptide profile of the diets was performed by size exclusion chromatography (SEC-HPLC) with two columns (600 kDa–20 kDa, <20 kDa). From each experimental diet, 2 g was acquired in duplicate.

### 2.4. Statistical Analysis

Significant differences in skeletal deformities rates among the diets and within each regime were tested with G-test [49]. Growth and survival differences were tested by means of Kruskal–Wallis and Mann–Whitney U-statistic. In the case of P12 diet of the first replicate at the DF regime, its growth and survival measurements were excluded from the mean rates due to accidental fish loss. At 15 dpf, the differences between the diets in gene expression within each regime were tested by one-way Anova. Factorial Anova followed by Tukey’s post-hoc tests determined the statistically significant differences in gene expression under the effect of the diets (C, P6, P12) and the external morphology (N, S) within each regime. The *p* < 0.05 level of significance was applied for all the statistical analyses.

## 3. Results

### 3.1. Peptide Profile and Free Amino Acid Content

**Figure 2:**
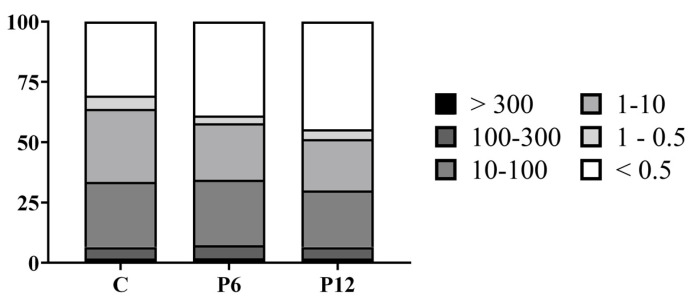
Peptide profile (%) of the experimental diets (C, P6, P12). Differences in molecular weight are presented in classes of kDa.

The peptide profile (%) of the feeds was evaluated by means of SEC-HPLC (Figure 2). The P12 diet had higher proportion (44.6%) of peptides with a molecular size of maximum 500 Da than P6 (38.9) and C (30.7), indicating an increased presence of di- and tripeptides. Total free amino acid content analysis revealed an increase in both essential (EAA) and non-essential (non-EAA) amino acids in the peptide diets (Table 2).

### 3.2. Exclusive Provision of Peptide Diets Is Beneficial for Survival and Growth

**Figure 3:**
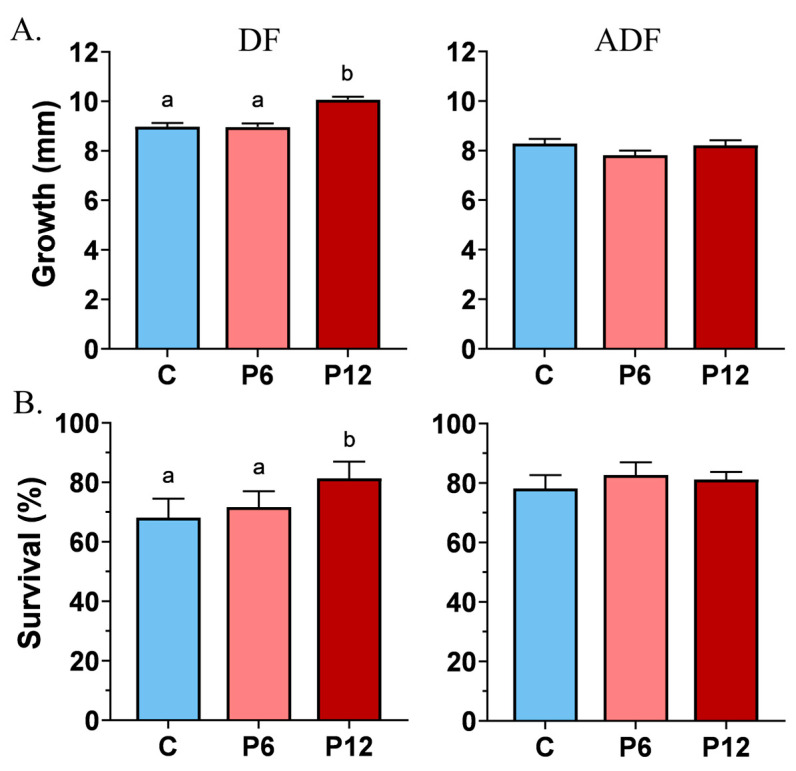
Effect of peptide diets (C, P6, P12) on the mean growth (±SE) (**A**) and survival (±SE) (**B**) at the end of the larval stage in the two feeding regimes (DF, ADF). Statistically significant differences between the diets are indicated by the absence of a common letter (*p* < 0.05).

Growth measurements before the SCT test revealed a shift towards a significantly increased TL mean of 10.1 ± 0.1 mm in the P12 group compared to P6 and C (9.0 ± 0.1 in both cases) in the absence of co-feeding (DF) (Figure 3A, DF). Artemia implementation (ADF) prevailed over any intergroup differences on growth (C: 8.3 ± 0.2, P6: 7.8 ± 0.2, P12: 8.0 ± 0.2) (Figure 3A, ADF).

Feeding regime DF highlighted also that peptide diet P12 induce a statistically significant higher survival rate reaching 81.4 ± 5.6%, compared with P6 (71.7 ± 5.3%) and C (68.1 ± 6.3%) (Figure 3B, DF). Concerning the ADF trial, no differences on survival rates were observed between the diets, whereas the range of the survival frequencies varied from 78.2 ± 2.6% (P6) to 81 ± 1.4% (P12) and 82.7 ± 2.5% (C) (Figure 3B, ADF).

No significant replicate effects were observed for both growth and survival rates in the two feeding regimes (Supplementary Figure S1).

### 3.3. P12 Diet Reduces the Abnormalities Rates When Dry Feeds Are the Sole Feeding Source

**Figure 4:**
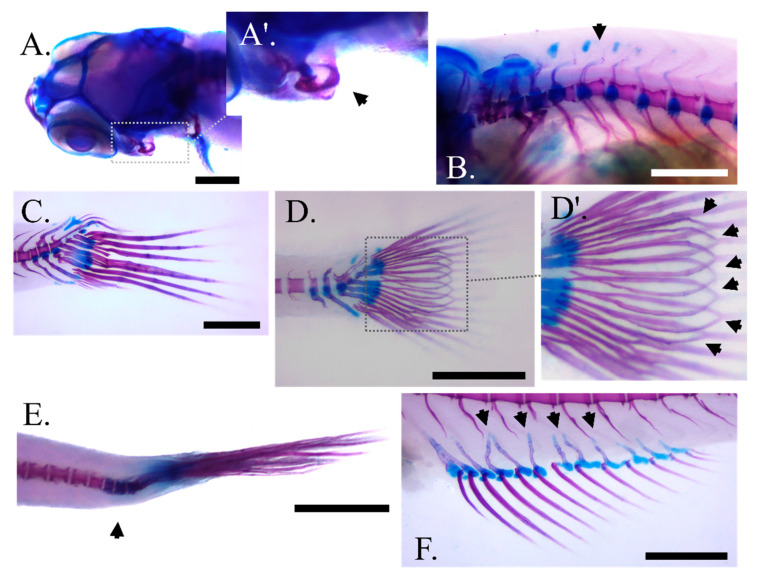
Main skeletal abnormality types observed at the end of larval rearing in zebrafish fed with the experimental diets (C, P6, P12) according to the two feeding regimes (DF, ADF). (**A**,**A′**) Angled view of the abnormal gill cover with magnification on the malformed bone, respectively. (**B**) Prehaemal kyphosis. (**C**) Missing and compressed caudal fin rays in DF regime. (**D**,**D′**) Unbundling of the caudal fin lepidotrichia in ADF regime with magnification on the malformed bones respectively (black arrows). (**E**) Scoliosis of the caudal peduncle. (**F**) Disorientated anal fin pterygiophores (black arrows). Scale bar equals 0.5 mm.

The most frequent abnormality types observed in our samples include an inner folding of the gill-cover (Figure 4A,A′), prehaemal kyphosis (Figure 4B), missing and compressed caudal fin rays (Figure 4C) or a between the lepidotrichia hemirays split accompanied by poor lepidotrichia ossification (Figure 4D,D′). Prehaemal kyphosis and caudal-peduncle scoliosis (Figure 4E) prevailed over the vertebrae deformities. Additionally, an abnormal formation of anal pterygiophores (Figure 4F) was observed.

**Figure 5:**
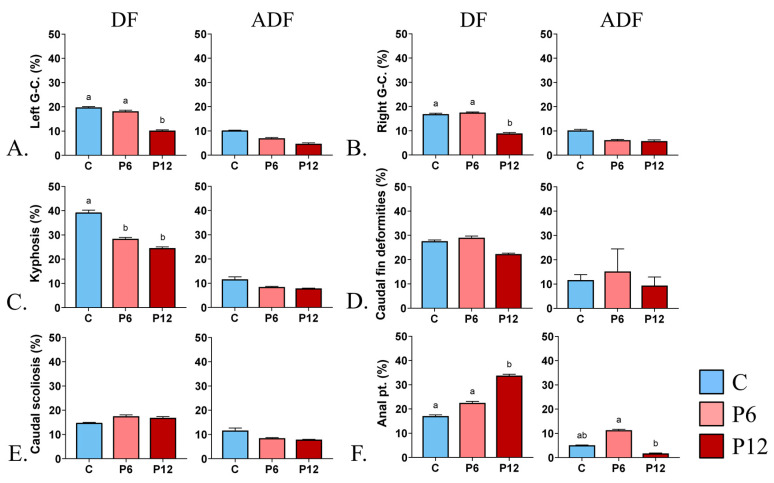
Effect of peptide diets (C, P6, P12) and feeding regimes (DF, ADF) on skeletal abnormalities frequency at the end of the larval period. (**A**) Abnormal left gill cover. (**B**) Abnormal right gill cover. (**C**) Prehaemal kyphosis. (**D**-DF) Missing and compressed caudal fin rays in DF regime. (**D**-ADF) Unbundling of the caudal fin lepidotrichia in ADF regime. (**E**) Scoliosis of the caudal peduncle (**F**) Abnormal proximal pterygiophores of the anal fin. Statistically significant differences between the diets are indicated by the absence of a common letter (*p* < 0.05). Error bars equal to 1 s.e. Prx, proximal pterygiophores. G-C., gill cover. Pt, pterygiophores.

DF highlighted the beneficial effect of P12 on the gill cover abnormalities (Left gill cover, 10.2 ± 0.3 and Right gill cover, 8.9 ± 0.4) compared with P6 (L, 18.2 ± 0.4 and R, 17.5 ± 0.3) and C (L, 19.8 ± 0.3 and R, 16.9 ± 0.3) (Figure 5A,B, DF). In addition, both P6 and P12 of the same regime resulted in lower kyphosis incidents, 28.4 ± 0.5 and 24.6 ± 0.5, respectively, in contrast with the C diet (39.3 ± 0.9) (Figure 5C, DF). Under the same DF regime, increased number of individuals with abnormal anal pterygiophores were reported in P12 (33.8 ± 0.6) (Figure 5F, DF). On the other hand, feeding regime ADF led to decreased rates of below 15% concerning every abnormality type regardless of the diet (Figure 5A–F, ADF).

### 3.4. Early Provision of Peptide Diets Reduced Haemal Lordosis Induced by SCT

**Figure 6:**
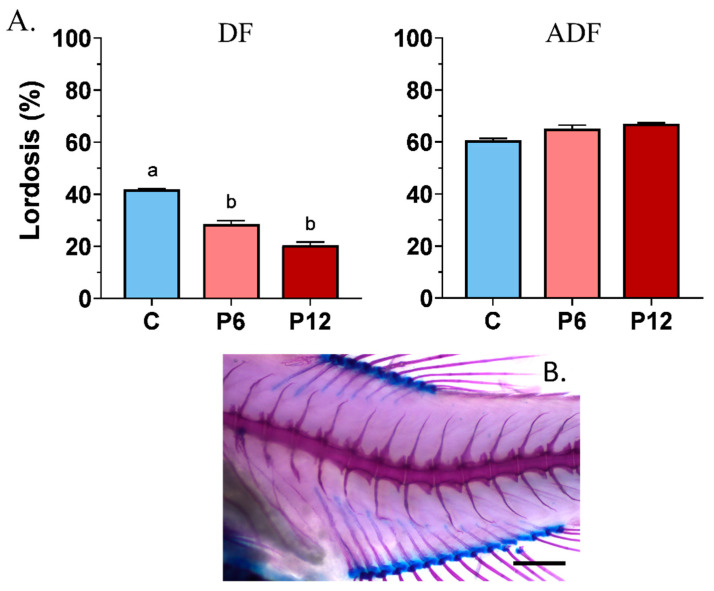
Frequency of haemal lordosis (**A**) after the SCT between the experimental diets (C, P6, P12) among the two feeding regimes tested (DF, ADF). A representative case of haemal lordosis is presented below the graphs (**B**). Statistically significant differences between the diets are indicated by the absence of a common letter (*p* < 0.05). Error bars equal to 1 s.e. Scale bar equals 0.5 mm.

Results showed that peptide diets, when provided from first feeding to zebrafish larvae (DF), can be beneficial in reducing the haemal lordotic incidents at 28.6 ± 1.3% (P6) and 19.4 ± 1.2% (P12) compared with the control one (C, 41.9 ± 0.2%) (Figure 6A, DF). On the contrary, a small inclusion of live food during the early stages (ADF) increased the haemal lordosis frequency to 60–70%, regardless of the presence of the shrimp peptides in the diet (Figure 6A, ADF). In both feeding regimes, no statistically significant differences existed among the replicates (*p* > 0.05).

### 3.5. No Significant Effect of the Peptide Diets on the Gene Expression during Larval Stage (15 dpf)

**Figure 7:**
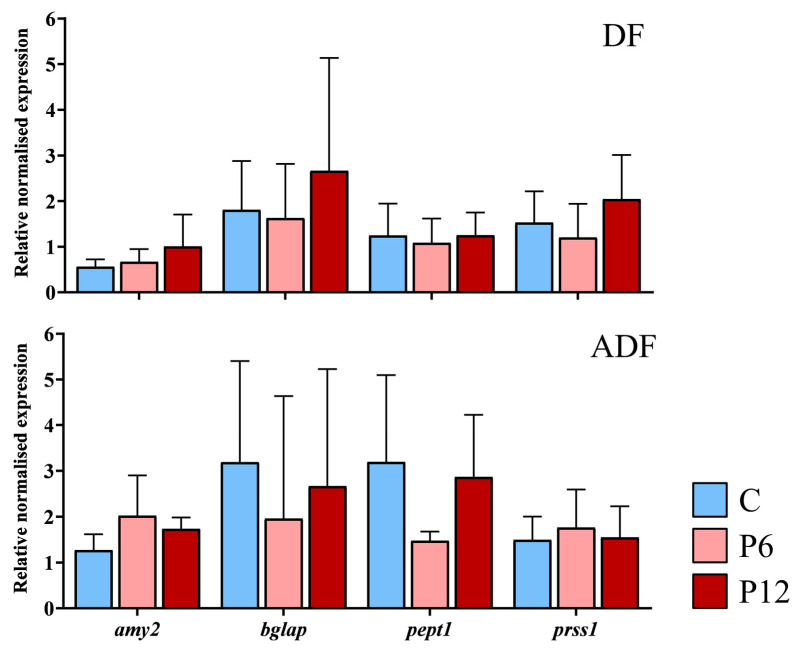
Relative expression of the amylase alpha 2a (amy2), osteocalcin (bglap), peptide transporter 1a (pept1) and trypsin (prss1) during the development (15 dpf) of the zebrafish larvae fed with the three experimental diets (C, P6, P12) according to the two regimes (DF, ADF). Values are presented as means ± SD. Statistically significant differences between the diets are indicated by the absence of a common letter (*n* = 4–8 pooled samples of 5 individuals per diet and gene, *p* < 0.05).

RT-qPCR assays during zebrafish early larval stage were aiming in examination of possible differential transcript expression between the experimental diets of gene involved in ossification status (*bglap*), peptide absorption (*pept1*) and gut function (*amy2a*, *prss1*). Results revealed no statistically significant differences in the mean expression of the selected genes among the diets, independently of the regime (Figure 7, DF and ADF, *p* > 0.05). No significant differences in gene expression were observed among the replicates within each diet and regime (*p* > 0.05).

### 3.6. Gene Expression after SCT Is Affected by the Diets and Feeding Regimes

**Figure 8:**
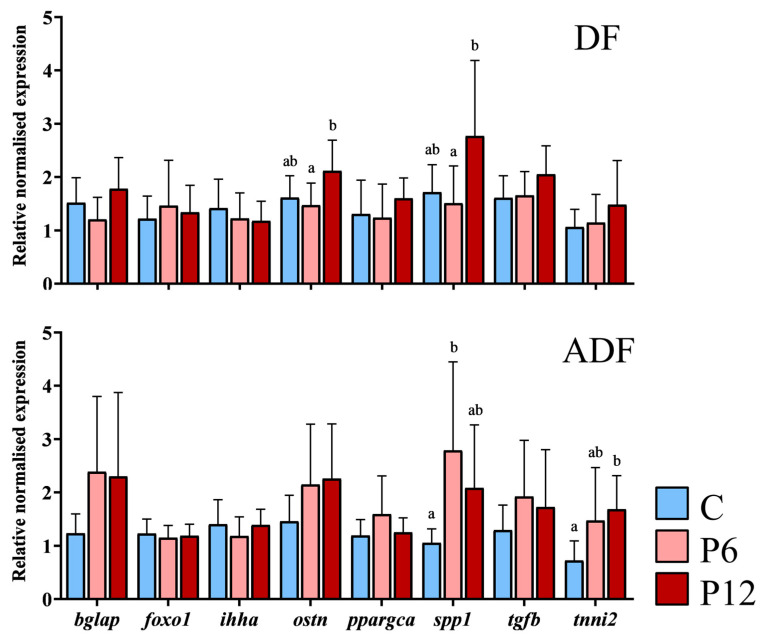
Relative expression of osteocalcin (bglap), forkhead box O1 a (foxo1a), Indian hedgehog signaling molecule a (ihha), osteocrin (ostn), peroxisome proliferator-activated receptor gamma coactivator 1 alpha (ppargca), osteopontin (spp1), transforming growth factor beta 1b (tgfb1b) and troponin I type 2a (tnni2a) after the SCT, between the experimental diets (C, P6, P12) and according to feeding regimes (DF and ADF). Values are presented as means ± SD. Statistically significant differences between the diets are indicated by the absence of a common letter (*n* = 3–6 pooled samples of three individuals per diet and gene, *p* < 0.05).

Regardless of the feeding regime, results highlighted that external phenotype (N~S) within each diet is not associated with any differential gene expression after the SCT (*p* > 0.05, Supplementary Table S4). Expression levels of three genes were influenced by the experimental diets (C, P6, P12) in the two feeding regimes (DF, ADF). *Spp1* transcript levels were significantly elevated in P12 diet (2.77 ± 1.42) in case of early diet provision (Figure 8, DF) compared with P6 (1.51 ± 0.70) but not compared with C (1.72 ± 0.51) diets. *Ostn* transcript levels also reached significantly higher levels in P12 (2.12 ± 0.57) compared with P6 (1.50 ± 0.41) but not C group (1.61 ± 0.41) under the same regime (Figure 8, DF). On the ADF regime (Figure 8, ADF), P6 presented the highest levels of *spp1* (2.79 ± 1.76), being statistically different from the C (1.05 ± 0.26) but not the P12 group (2.08 ± 1.18). Simultaneously, *tnni2* presented a significantly higher expression in the P12 group (1.68 ± 0.63) compared to the C but not the P6 (0.72 ± 0.37 and 1.47 ± 1.00 respectively, Figure 8, ADF) group.

## 4. Discussion

Total diet evaluation revealed a positive effect of short peptide incorporation in zebrafish rearing providing their availability from first feeding. Total larval abnormality rates decrease when smaller protein fractions (e.g., hydrolysates, peptides) are included in their nutrition [9,19,25]. In agreement with this, our results of the larval quality control recorded at the end of metamorphosis report a positive impact of P12 diet on gill cover abnormalities and pre-haemal kyphosis given that the artificial diets are the sole feeding source. Interestingly, an association of kyphosis incidents with the frequency of deformities in branchiostegal rays on sea bass larvae highlighted the common ossification pattern (membranous bones) and timing of appearance between the gill cover and the first pre-haemal vertebrae bodies [50,51]. Specifically, the operculum complex and the pre-haemal vertebral centra belong to the skeletal characters whose ossification can be detected even on a 4 mm (Standard Length—SL) zebrafish larvae [46,52]. However, if we take into consideration the reverse effect of P12 in anal pterygiophores abnormalities frequency, which form typically after 7 mm SL in zebrafish as endochondral bones [46,53], an early peptide-enhanced ossification scenario is suggested. A possible peptide-driven advanced intestinal digestion could potentially promote fish harmonious skeletal development through better absorption of skeletogenesis-related nutrients [15]. Being a stomachless species, zebrafish pancreatic enzymatic secretion is mainly indicative of digestion with endoproteases (e.g., trypsin) and saccharidase (e.g., amylase) being detected from 7 dpf [35]. Our results, though, do not report any significant changes in the relative expression of amylase and trypsin at 15 dpf. Similarly, no effect of the peptide diets was detected on the expression of the brush border membrane proton/oligopeptide transporter pept1. Therefore, a peptide-enhanced total larval maturation as previously described on marine fish larvae [21] cannot be confirmed from the present study. An earlier sampling within the first days of exogenous feeding would potentially allow the detection of possible differences related with the maturation of the digestive system eliminating any possible ontogenetic effect within each sample. Concerning the ADF regime, the diets did not present any significant effect on the abnormalities rates, with the total frequencies ranging below 15%. This is in accordance with the literature, stating that live-food incorporation during the early stages favors the normal zebrafish skeletal development of the larvae cephalic region and vertebral column [8].

A beneficial effect of the peptide diets was also highlighted after the SCT under the DF regime, where the incidents of swimming-induced haemal lordosis were reduced in the peptide groups (P6, P12). The swimming-induced lordosis frequency is a valuable means of testing the vulnerability of a normally ossified vertebral column and its surrounding muscle against increased swimming conditions [5,43]. Focusing on the center of the deformity, the haemal vertebrae centra with their haemal and neural arches, a correlation between increased light deformations in the spines prior to the swimming test and increased frequency of lordosis induction after has already been described [4]. Given the common membranous origin of the spines, a differential ossification pattern between the diets could be also proposed. Even though osteocalcin expression did not present any changes among our experimental groups before and after the SCT, elevated expression of *spp1* and *ostn* in P12 juvenile group could suggest a differential regulation of mineralization during the SCT. Both proteins are present in osteoblast ECM and are in charge of the attachment of osteoblasts and osteoclasts to the bone matrix [54]. Thus, the increased resistance of P12 individuals against the intense swimming could be attributed to a more efficient bone mineralization of the haemal region. This hypothesis is in accordance with previous literature reporting increasing bone resistance to fractures through an increased mineralization pattern [31]. A fluorescent follow-up of the calcified skeletal elements during the osteogenesis could be necessary to determine a potentially deferential peptide-driven ossification pattern. Surprisingly, in the ADF case, haemal lordosis frequency reached 60.7–67.1% regardless of the dietary groups. This observation could be attributed to a larval preference of live-prey over dry feeding during the initial co-feeding period [55,56]. This preference could potentially trigger a differential musculoskeletal response during the SCT (*spp1* and *tnni2* increased expression in P6 and P12 diets, respectively, after the SCT in ADF regime), explaining the increased lordosis counts in this regime. Additionally, since lordosis induction requires muscular adaptations of the haemal region [34,57], future research should focus on the effect of early nutrition on larval and post-larval musculoskeletal development.

Protein hydrolysates of different sources and levels of incorporation have been proven beneficial for early larvae growth and survival in sea bass and sea bream [24,25]. Our study, for the first time, reports a similar result in zebrafish larvae when the peptides diets are provided exclusively throughout the rearing. When live-food is not included in the regime, a 12% substitution of the total dietary protein with shrimp di and tripeptides led to a significant increase in growth and survival at the end of the larval period. Since the 6% of substitution group was not differentiated from the control, we assume a dose-dependent peptide effect as previously described for sea bass (around 20% of hydrolysate incorporation in the total protein fraction as optimum [21,24]) and sea bream (around 9% [25]). However, a short live-food inclusion (ADF) equalizes any intergroup differences in terms of growth and survival. Up to now, knowledge reports that rearing zebrafish with artificial diets from first feeding is feasible, compromising with either lower survival or growth rates compared to a live-food rearing [58]. A possibility of significant performance rates can only be achieved by continuous provision of an appropriate early diet [59]. Despite the limitations owed to the non-common origin of the populations between the feeding regimes, our study presents the first evidence that zebrafish larvae can reach high growth and survival rates either under an exclusive non-continuous dry diet regime or a co-feeding protocol with Artemia nauplii. Therefore, an early diet with efficient characteristics (e.g., size, solubility, sinking rate, color, leaching rate, biochemical composition and ingredient bioavailability, and digestibility) and distribution during the critical period after mouth opening could potentially replace effectively the live preys [23,60].

Undoubtedly, the differential free amino acid profile induced by the peptide incorporation on our diets cannot be overlooked. Skeletogenesis can be affected by a differentiated amino acid (AA) profile [25]. Among the essential amino acids, methionine is known to be necessary for growth and feed utilization in fish larvae [61]. Under a species-specific effect, a tryptophan deficiency has been correlated with increased scoliosis incidents in salmonids [20] whereas no similar effect was observed in a zebrafish species [62]. Moreover, glutamate, leucine and glutamine have been suggested as the preferred nutrient substrates to generate ATP in zebrafish skeletal muscle [63,64]. Furthermore, a lysine deficiency can regulate muscle growth in the species [65]. Given this background, the differences in methionine, leucine and lysine between the peptide diets and our control one could potentially induce an altered muscle developmental pattern from early stages. Based on the musculoskeletal nature of the haemal lordosis [34], a potential either enforcement or myopathy induced by the peptides could explain the differential response and/or ability to adapt under increased swimming conditions.

Presently, several trials attempting to estimate the dietary protein and amino acid requirements of zebrafish have highlighted the possibility for rearing the species with exclusive provision of formulated diets [66]. With the present study, we not only confirm this assumption but we suggest the use of peptides for controlling the larval and post-larval skeletal development. Further research is required to clarify the exact molecular pathway of peptide effect on the musculoskeletal system. Nevertheless, zebrafish can successfully be utilized as a model species for nutritional genomics studies, shedding light on similar processes in aquaculture fish [67].

## Figures and Tables

**Table 1:** Formulation and proximate composition of the experimental diets (C, P6, P12).

Ingredients (g kg^−1^, Dry Matter Basis)	C	P6	P12
Fish meal ^a^ (protein 74.7%, lipids 7.5%, EPA + DHA 20.9%)	640	670	620
Shrimp peptides (protein 68.4%, lipids 8.6%, EPA + DHA 1.1%)	0	50	100
CP SP 90 (protein 88.2%, lipids 2.5%, EPA + DHA 18.1%)	130	50	50
Fish oil ^b^	10	10	10
Rape seed lecithin ^c^	130	130	130
Starch	50	50	50
Vitamin mix ^d^	30	30	30
Mineral mix ^e^	10	10	10
Theoretical composition (%)			
Protein Peptide/protein ratio	54.7 0.0	53.2 6.1	53.0 12.3
Lipids	16.7	17.1	17.9
EPA + DHA	1.1	1.2	1.1

^a,b^ Fish meal and oil were provided from La Lorientaise (Lorient, France). ^c^ SAIPOL, Grand Couronne, France. ^d^ Composition per kg of vitamin mix: retinyl acetate, 340 mg; cholecalciferol, 2.5 mg; all-rac-α-tocopherol acetate, 4 g; menadione, 0.1 g; thiamin, 1 g; riboflavine, 2.5 g; D-calcium pantothenate, 5 g; pyridoxine HCl, 1 g; cyanocobalamin, 0.006 g; niacin, 10 mg; folic acid, 0.5 g; biotine, 0.1 g; meso-inositol, 100 g. ^e^ Composition per kg of mineral mix: KCl, 90 g; KI, 40 mg; CaHPO_4_·2H_2_O, 500 g; NaCl, 40 g; CuSO_4_·5H_2_O, 3 g; ZnSO_4_·7H_2_O, 4 g; CoSO_4_·7H_2_O, 20 mg; FeSO_4_·7H_2_O, 20 g; MnSO_4_·H_2_O, 3 g; CaCO_3_, 215 g; MgSO_4_·7H_2_O, 124 g; NaF, 1 g.

**Table 2:** Free amino-acid composition of the experimental diets (%). EAA, essential amino acids. Sum free AA, summary of free amino acids.

Amino Acids (% Feed)		C	P6	P12
EAA	Threonine	0.04	0.1	0.14
	Valine	0.05	0.13	0.19
	Methionine	0.02	0.06	0.09
	Isoleucine	0.04	0.11	0.16
	Leucine	0.08	0.2	0.29
	Phenylalanine	0.04	0.13	0.2
	Histidine	<0.02	0.03	0.05
	Lysine	0.05	0.19	0.29
non EAA	Aspartic acid	0.03	0.09	0.13
	Serine	0.02	0.06	0.09
	Glutamic acid	0.07	0.16	0.22
	Proline	0.03	0.11	0.18
	Glycine	0.05	0.13	0.19
	Alanine	0.1	0.2	0.27
	Cystine	<0.02	<0.02	<0.02
	Tyrosine	0.03	0.11	0.18
	Arginine	0.04	0.19	0.32
Sum free AA		0.69	2.00	2.99

## Data Availability

The data can be made available upon reasonable request to the authors.

## Supplementary Materials

The following supporting information can be downloaded at: https://www.mdpi.com/article/10.3390/biom13040659/s1. Table S1. Mean values (± SD) of the abiotic parameters followed throughout the experiment. Oxygen saturation was measured in each net pen whereas for the rest parameters one common measure was made on the water outside the pens. Table S2. Feeding quantities and frequency of the two feeding regimes (DF—dry feed only, ADF—Artemia and dry feed) applied among the experimental diets (C, P6, P12). Dpf, days post fertilization. Table S3. Accession numbers and nucleotide sequences of the primers used for the RT-PCR. *, existence of more than one accession number covers the possibility of more than one transcript variants included. Figure S1. Effect of peptide diets (C, P6, P12) on growth (A) and survival (B) in the different replicates (Repl.1–Repl.3) at the end of the experimental trials under the DF and ADF feeding regimes respectively. #, indicates the accidental loss of larvae in P12, replicate one, ADF. This group was excluded from the mean survival estimation. Statistically significant differences between the diets are indicated by the absence of a common letter (*p* < 0.05). Table S4. Summary of statistical analysis on gene expression data. The diet predictor refers to the C, P6 or P12. The phenotype predictor within each diet refer to the external categorization in lordotic (S) or normal (N). Statistical significant differences (*p* < 0.05) are indicated with the asterisks (**). SS, Sum of Squares. df, degrees of freedom. MS, Mean Square.
