# Supplementary material for: Effect of Early Peptide Diets on Zebrafish Skeletal Development
Source: Biomolecules. 2023 Apr 7;13(4):659. doi: 10.3390/biom13040659 (PMC10135642; doi:10.3390/biom13040659)
Supplement: Supplementary file 1 [file biomolecules-13-00659-s001.zip › biomolecules-2298168-supplementary.pdf]

Effect of Early Peptide Diets on Zebrafish Skeletal Development

A. Printzi [1,2,*], G. Koumoundouros [1], V. Fournier [3], L. Madec [2], J.-L. Zambonino-Infante [2] and D. Mazurais [2]

[1] Biology Department, University of Crete, 70013 Crete, Greece
[2] IFREMER, University of Brest, CNRS, IRD, LEMAR, F-29280 Plouzané, France
[3] DianaAqua, Symrise Group, 56250 Elven, France
* Correspondence: aprintzi@ifremer.fr

Table S1. Mean values (± SD) of the abiotic parameters followed throughout the experiment. Oxygen saturation was measured in each net pen whereas for the rest parameters one common measure was made on the water outside the pens.

Parameters	Trial	Condition	Replicate I	Replicate II	Replicate III
Temperature (°C)	1	all	27.9 ± 0.3	27.9 ± 0.4	28.2 ± 0.2
	2	all	28.3 ± 0.4	28.2 ± 0.4	28.1 ± 0.3
pH	1	all	7.6 ± 0.7	7.5 ± 0.4	8.2 ± 0.0
	2	all	8.2 ± 0.1	8.2 ± 0.1	8.2 ± 0.1
Conductivity (μS/cm)	1	all	464 ± 26	492 ± 40	484 ± 16
	2	all	486 ± 24	512 ± 48	478 ± 10
Ammonia (mg/L)	1	all	<0.01	<0.01	<0.01
	2	all	<0.01	<0.01	<0.01
Nitrate (mg/L)	1	all	2.67 ± 2.08	1.01 ± 0.99	1.50 ± 0.71
	2	all	1.50 ± 0.71	2.75 ± 1.06	2.17 ± 1.26
Nitrite (mg/L)	1	all	0.030 ± 0.017	0.007 ± 0.012	0.013 ± 0.012
	2	all	0.001 ± 0.014	0.012 ± 0.012	0.013 ± 0.012
O_2 concentration (mg/L)	1	C	6.6 ± 0.2	7.3 ± 0.3	6.7 ± 0.8
		P6	6.5 ± 0.2	7.0 ± 0.4	6.8 ± 0.6
		P12	6.8 ± 0.7	7.1 ± 0.3	7.0 ± 0.5
	2	C	6.5 ± 1.1	6.7 ± 0.4	7.1 ± 0.5
		P6	6.9 ± 0.9	6.7 ± 0.5	7.3 ± 0.4
		P12	7.1 ± 0.9	6.9 ± 0.5	7.1 ± 0.5

Table S1. Feeding quantities and frequency of the two feeding regimes (DF - dry feed only, ADF – Artemia and dry feed) applied among the experimental diets (C, P6, P12). Dpf, days post fertilization.

dpf	Feed	Trial	C, P6, P12

			Quantity/meal	Meals/day
5-7	Artemia nau.	DF	-	-
		ADF	4000	5
	df (mg)	DF	50	5
		ADF	-	-
8-11	Artemia nau.	DF	-	-
		ADF	8000	3
	df (mg)	DF	75	5
		ADF	50	2
12-16	Artemia nau.	DF	-	-
		ADF	-	-
	df (mg)	DF	75	5
		ADF	75	5
17-22	Artemia nau.	DF	-	-
		ADF	-	-
	df (mg)	DF	100	5
		ADF	100	5
23-	Artemia nau.	DF	-	-
		ADF	-	-
	df (mg)	DF	150	5
		ADF	150	5

Table S2. Accession numbers and nucleotide sequences of the primers used for the RT-PCR. *, existence of more than one accession number covers the possibility of more than one transcript variants included.

Gene (Accession no.)	Forward primer (5′ – 3′)	Reverse primer (5′ – 3′)
Bglap (NM_001083857.3)	CAGCTGACACAGAAGCGAAC	AGGAGTCAGGAAGACCTGCG
Pept1 (NM_198064.1)	TTCGGACAAACTTGCAGCGA	ACACCACTTCACCACAGGTC
Amy2a (NM_213011.2)	CCAACACCAAAAGCGGAAGG	TCACTTGGAGGGGAGATCTGA

Prss1 (NM_131708.2)	TCTCTCTGCCTTCAAGCTGC	CGGCTTGGGTAATTGCTTCC
Ppargc1a (XM_017357139.2, XM_002667531.6) *	AGAGAACTAGAGTGCGCTGC	AGAAAGCTATCCGCGTCGAG
Ppargc1b (XM_009291061.3)	GGCTTCTTTTCCTAGCCGGT	AGGAGCGTTTCTCGTCACTG
Ostn (NM_001326409.1)	GACCTTTTGGCCCTGGACTT	TGTGGGACAGGAACCCATCA
Tnni2a (NM_001007365.1)	TGCCTCAGCTTATAGGGGCAA	AACCCTTAGCGATGGAGAGC
Spp1 (NM_001002308.1)	GTTTTAACACTCCTCGTCGCC	GAAGAATAGGAGGTGGCCGTT
Ihha (NM_001034993.2)	CAGTGGAGGCCGGTTTTGAT	AGCCACAGAGTGCTCTGACTT
Foxo1a (NM_001077257.2)	CGGCAAAGAAAAAGCTGGCT C	GACGACCGCTTAATGTGCTG
Tgfb1b (XM_687246.8)	TGTGACGTCCACCCTGAAAG	ATTGCGGGACAAACCTGCTA
Ef1 (NM_131263.1)	CTTCTCAGGCTGACTGTGC	CCGCTAGCATTACCCTCC
Actn2b (NM_181601.5)	AATTGCCGCACTGGTTGTTG	ACCAACCATGACACCCTGATG

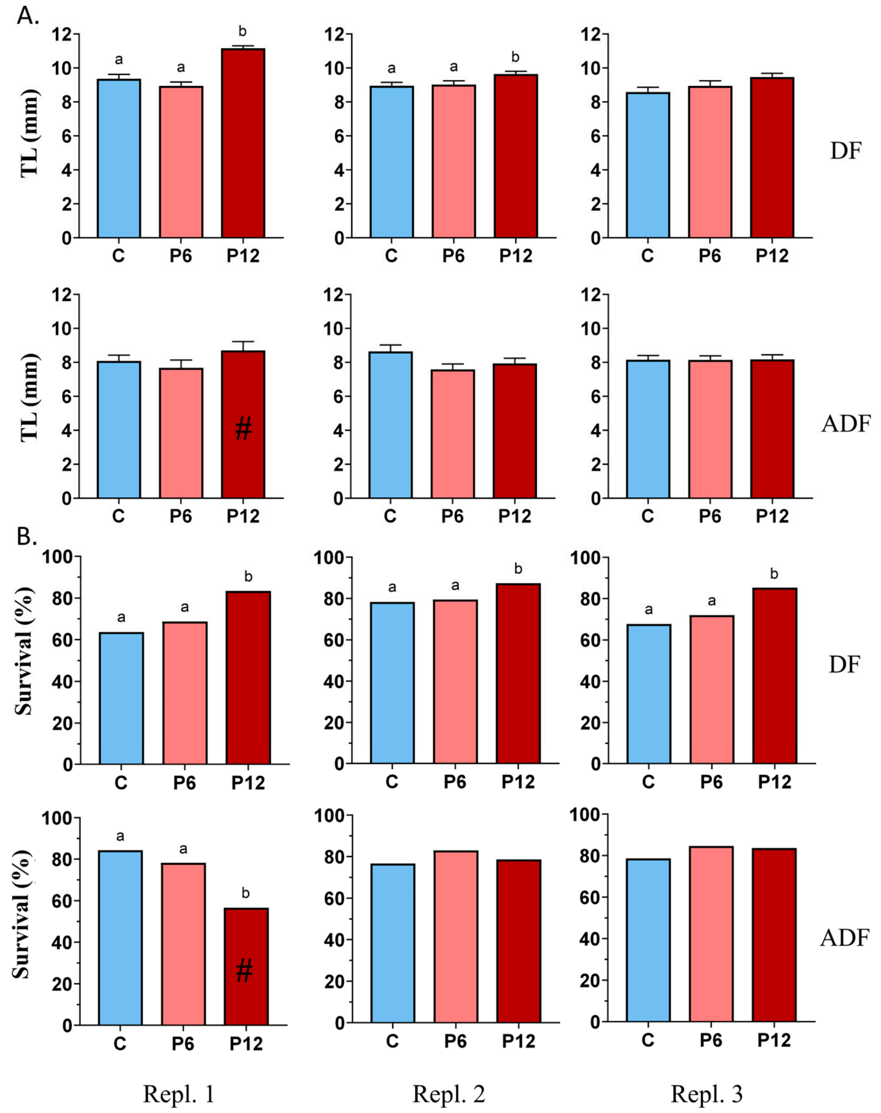

Figure S1. Effect of peptide diets (C, P6, P12) on growth (A) and survival (B) in the different replicates (Repl.1-Repl.3) at the end of the experimental trials under the DF and ADF feeding regimes respectively. #, indicates the accidental loss of larvae in P12, replicate one, ADF. This group was excluded from the mean survival estimation. Statistically significant differences between the diets are indicated by the absence of a common letter ($p < 0.05$).

Table S4. Summary of statistical analysis on gene expression data. The diet predictor refers to the C, P6 or P12. The phenotype predictor within each diet refer to the external categorization in lordotic (S) or normal (N). Statistical significant differences ($p<0.05$) are indicated with the asterisks (**). SS, Sum of Squares. df, degrees of freedom. MS, Mean Square.

Gene	Regime	Predictor	SS	df	MS	F	p
bglap	DF	Diet	1.33	2	0.66	2.58	0.10
		Phenotype (diet)	0.03	1	0.03	0.11	0.74
	ADF	Diet	8.14	2	4.07	2.80	0.08
		Phenotype (diet)	0.55	1	0.55	0.38	0.55
foxo1	DF	Diet	0.25	2	0.13	0.45	0.64
		Phenotype (diet)	1.12	1	1.12	4.01	0.06
	ADF	Diet	0.01	2	0.00	0.07	0.94
		Phenotype (diet)	0.11	1	0.11	1.69	0.21

ihha	DF	Diet	0.30	2	0.15	0.74	0.49
		Phenotype (diet)	0.20	1	0.20	0.97	0.34
	ADF	Diet	0.29	2	0.14	0.92	0.42
		Phenotype (diet)	0.44	1	0.44	2.82	0.11
ostn	DF	Diet	1.87	2	0.93	3.80	**
		Phenotype (diet)	0.00	1	0.00	0.00	0.95
	ADF	Diet	3.98	2	1.99	2.35	0.12
		Phenotype (diet)	1.05	1	1.05	1.24	0.28
pparg	DF	Diet	0.60	2	0.30	1.02	0.38
		Phenotype (diet)	0.45	1	0.45	1.51	0.23
	ADF	Diet	0.85	2	0.43	1.67	0.21
		Phenotype (diet)	0.25	1	0.25	0.99	0.33
spp1	DF	Diet	7.37	2	3.68	4.32	**
		Phenotype (diet)	2.17	1	2.17	2.54	0.13
	ADF	Diet	15.30	2	7.65	5.63	**
		Phenotype (diet)	1.07	1	1.07	0.79	0.38
tgfb	DF	Diet	0.96	2	0.48	2.21	0.13
		Phenotype (diet)	0.08	1	0.08	0.36	0.56
	ADF	Diet	2.42	2	1.21	1.51	0.25
		Phenotype (diet)	0.29	1	0.29	0.37	0.55
tnni2	DF	Diet	0.80	2	0.40	1.20	0.32
		Phenotype (diet)	0.20	1	0.20	0.61	0.44
	ADF	Diet	4.10	2	2.05	4.06	**
		Phenotype (diet)	0.47	1	0.47	0.92	0.35
